# The Early Impact of the IDEA Collaboration Results: How the Results Changed Prescribing Practice

**DOI:** 10.1093/jncics/pkab043

**Published:** 2021-06-15

**Authors:** Timothy Iveson, Catherine Hanna, Poppy Iveson, Sui Zhang, Alexandra Levasseur, Jeffrey Meyerhardt

**Affiliations:** 1 University of Southampton, Southampton, UK; 2 CRUK Clinical Trials Unit, Glasgow, Scotland; 3 University of Oxford, Oxford, UK; 4 Dana-Farber Cancer Institute, Boston, MA, USA; 5 Alliance for Clinical Trials in Oncology, Chicago, IL, USA

## Abstract

**Background:**

Traditionally, adjuvant treatment for colon cancer has been 6 months of combination chemotherapy. Six phase III trials tested the hypothesis that 3 months is noninferior in efficacy to 6 months and reduces long-term side effects for patients. The results were pooled in the International Duration Evaluation of Adjuvant therapy (IDEA) collaboration. Although this did not meet the noninferiority endpoint, a preplanned subgroup analysis by chemotherapy regimen did demonstrate noninferiority for capecitabine and oxaliplatin. Additionally, risk stratification by T and N stage was defined.

**Methods:**

In an effort to understand the real-life impact of these results, 4 months after the IDEA results, an online survey was distributed to clinicians to ask their approach to the adjuvant treatment of patients with stage III colon cancer.

**Results:**

The survey was completed by 458 clinicians from 12 countries. Assuming that 6 months of treatment was the pretrial standard of care, 89.5% of clinicians reported they had changed practice to prescribe 3 months of treatment for some patients. For patients with low-risk stage III disease, there was a preference for 3 months, and for patients with high-risk stage III disease, most clinicians still prescribed 6 months at that time. Overall, capecitabine and oxaliplatin regimen was the most popular. There were important differences in responses depending on the location of respondent and T and N stage of disease.

**Conclusion:**

This survey shows that the IDEA collaboration has been practice changing but reveals important differences in the way results are interpreted by individual clinicians.

Six months of oxaliplatin-fluoropyrimidine chemotherapy has been a recommended adjuvant treatment for stage III colon cancer for more than a decade (1-3). This treatment confers a risk of permanent peripheral neuropathy, which can have long-lasting effects on patients’ quality of life (4). Recently, 6 individual clinical trials investigated the efficacy and toxicity of 3 months of treatment compared with the standard 6 months. In a unique, international effort, these trial results were analyzed together in the International Duration Evaluation of Adjuvant therapy (IDEA) collaboration (5). The results of this pooled analysis were presented at the annual meeting of the American Society of Clinical Oncology (ASCO) Plenary Session in June 2017 and published in full in March 2018 (6). Updated results were presented at ASCO 2020 and published in full in December 2020 (7).

The IDEA collaboration did not meet the prespecified test for noninferiority of 3 months of chemotherapy vs 6 months for the overall patient population. However, an unanticipated outcome from a preplanned subgroup analysis revealed that the results differed depending on the regimen prescribed. For patients prescribed capecitabine—the oral pro-drug of 5-fluorouracil—in combination with oxaliplatin (CAPOX), a duration of 3 months was noninferior to 6 months of treatment. However, for those prescribed intravenous 5-fluorouracil and oxaliplatin (FOLFOX), 6 months was superior to 3 months of treatment. In addition, exploratory analyses demonstrated noninferiority of the shorter treatment duration for patients with T1-3, N1 disease but not for patients with T4 and/or N2 disease. As a consequence of these analyses, risk stratification between patients with earlier stage disease (T1-3, N1), known as low-risk stage III, vs more advanced disease (T4 or N2 disease or both), known as high-risk stage III, is now recognized. Updated results showed there was minimal clinical difference in 5-year overall survival (OS) between the 3- vs 6-month arm, although noninferiority was still not met statistically; subgroup findings were maintained.

Of the individual studies that contributed to the IDEA collaboration, the results from 5 individual trials have reported on the primary endpoint of 3-year disease-free survival (DFS) for 3 months vs 6 months of treatment. The SCOT trial did meet its primary endpoint and demonstrated, in the overall population, 3-year DFS with 3 months of adjuvant doublet chemotherapy (76.7%, 95% confidence interval [CI] = 75.1 to 78.2) was noninferior to 6 months (77.1%, 95% CI = 75.6 to 78.6; hazard ratio [HR] = 1.006, 95% CI = 0.909 to 1.114; test for noninferiority *P* = .012) (8). The ACHIEVE trial has reported a 3-year DFS rate of 79.5% for 3 months and 77.9% for 6 months, with a hazard ratio of 0.954 (95% CI = 0.758 to 1.201). There were only 291 (23%) DFS events, not a sufficient number for this result to be powered to meet statistical significance (9). The HORG trial reported that 3-year DFS for patients receiving 3 months of treatment was 77.2% (95% CI = 72.1% to 82.3%) vs 77.9% (95% CI = 72.6% to 82.5%) for the 6-month arm (HR = 1.05, 95% CI = 0.61 to 1.55) (10). In the SCOT, ACHIEVE, and HORG trials, the majority of patients received treatment with the CAPOX regimen. The TOSCA trial, performed in Italy, failed to show that 3 months of doublet chemotherapy was noninferior to 6 months (11). The hazard ratio for relapse or death using 3 months compared with 6 months was 1.14 (95% CI = 0.99 to 1.31; *P* for noninferiority 0.253 and the CI crossed the prespecified limit of 1.20). Despite this, the actual difference in relapse-free survival rate was small (3% at 5 years). In the IDEA-France trial, an advantage for 6 months of treatment was seen, with a 3-year DFS of 72.0% in the 3-month arm and 76.0% in the 6-month arm (HR = 1.24, 95% CI = 1.05 to 1.46; *P* = .012) (12). In both the TOSCA and IDEA-France trials, the majority of patients received FOLFOX chemotherapy.

Given these unanticipated outcomes regarding regimen and disease risk stage, there was uncertainty around how the IDEA collaboration results would be used by the clinical community globally (13). The aim of this study was to explore the real-life impacts of the IDEA collaboration by assessing if, and how, clinicians incorporated these results into their routine practice. This paper describes the results of a survey that asked international clinicians about their prescribing practices 4 months after the first reporting of the outcomes of the IDEA collaboration at ASCO 2017.

## Methods

In October 2017, an online survey was disseminated to the coordinators of all trials that contributed data to the IDEA collaboration, with the intention that it was electronically distributed to principal investigators and their colleagues who treat colon cancer. The 6 trials were SCOT (participating countries included the United Kingdom, Denmark, Spain, New Zealand, Australia, Sweden), IDEA-France (France), HORG (Greece), ACHIEVE (Japan), CALGB/SWOG 80702 (United States and Canada), and TOSCA (Italy). The survey was open for 3 weeks.

The aim of the survey was to probe the clinicians’ interpretation of the IDEA collaboration results overall and the results from the exploratory analyses. The survey was comprised of 13 questions: 5 with a binary (yes/no) response and 8 clinical scenarios with a list of 6 prespecified possible answers. Section 1 included 5 questions that asked clinicians about their general approach to treating patients with stage III colon cancer. In section 2, participants were presented with 8 hypothetical patient scenarios. Table 1 shows the details of the disease stage for each of the 8 scenarios; 7 of these described patients with stage III colon cancer, and 1 described a patient with stage II colon cancer. This article presents the stage III scenario results only.

**Table 1.** Hypothetical patient scenarios included in section 2 of the survey^a^

Scenario No.	T stage	N stage	Differentiation	Age, y	IDEA collaboration defined “risk” group
1	1	1	Moderate	68	Low
2	4	1	Moderate	72	High (T4)
3	2	2	Well	60	High (N2)
4	3	1	Well	34	Low
5	3	1	Poor	54	Low
6	3	2	Moderate	62	High (N2)
7	4	2	Poor	74	High (T4 and N2)
8	4	0	Moderate	48	NA

a NA = not applicable. IDEA = International Duration Evaluation of Adjuvant therapy.

The survey was distributed 4 months after the initial presentation of the IDEA collaboration results and before full publication in peer-reviewed journals. Responses were collected using online software (SurveyMonkey) and analyzed using descriptive statistics in Microsoft Excel 2016.

## Results

### Survey Results

Overall, 458 clinicians responded to the survey. Three participants did not complete any questions after registration and were excluded from further analysis. The remaining participants were from Japan (54.3%), the United States (17.6%), the United Kingdom (10.1%), Italy (7.9%), Greece (3.5%), Australia (2.9%), Sweden (1.3%), Denmark (0.9%), New Zealand (0.4%), Germany (0.2%), France (0.2%), and Cyprus (0.4%). For 5 respondents, the country of residence was unknown (1.1%). For the purposes of this analysis, the countries were divided into 3 categories—United States and Canada; Europe and Other; and Japan—and will subsequently be referred to as the United States, Europe, and Japan. Approximately one-third of respondents worked in academic medical oncology (34.1%), one-third had a surgical background (33.1%), one-quarter (25.1%) described themselves as working primarily in health service medical oncology, and the remainder were radiation oncologists (2.6%) or identified as “other” (4.5%). The majority (144 of 152, 94.7%) of surgeons were from Japan, and it is routine for surgeons in Japan to prescribe adjuvant chemotherapy for patients with colon cancer.

### Overall Approach to Adjuvant Prescribing

Table 2 outlines respondents’ approach to treating patients with stage III colon cancer. The majority (89.5%) indicated they would prescribe 3 months of adjuvant chemotherapy for some patients. The majority of respondents from Japan (69.2%) and Europe (59.1%) felt that the interaction between duration of treatment and chemotherapy regimen prescribed reflected a difference in the clinical efficacy of CAPOX vs FOLFOX, whereas only a minority of clinicians from the United States agreed with this statement (28.8%). Most clinicians from all locations—the United States (91.2%), Europe (88.2%), and Japan (89.9%)—agreed with grouping patients with stage III colon cancer into high risk or low risk based on T and N staging.

**Table 2.** Respondents’ overall approach to adjuvant prescribing for patients with stage III colon cancer^a^

Survey question	Europe		USA		Japan		Overall	
	Yes, %	No, %	Yes, %	No, %	Yes, %	No, %	Yes, %	No, %
Should ALL patients with stage III colon cancer receive 6 months treatment?	9.4	90.6	12.5	87.5	24.0	76.0	17.7	82.3
Should ALL patients with stage III colon cancer receive 3 months treatment?	13.5	86.5	7.5	92.5	19.1	80.9	15.6	84.4
Do CAPOX and FOLFOX differ in their efficacy as adjuvant treatment in stage III colon cancer?	59.1	40.9	28.8	71.2	69.2	30.8	59.0	41.0
Should SOME patients with stage III colon cancer receive 3 months treatment?	94.5	5.5	91.2	8.8	86.2	13.8	89.5	10.5
Is dividing stage III colon cancer into low-risk and high-risk disease useful and clinically relevant?	88.2	11.8	91.2	8.8	89.9	10.1	89.3	10.7

^a^CAPOX = capecitabine oxaliplatin; FOLFOX = 5-fluorouracil and oxaliplatin; USA = United States.

### Clinical Scenarios

**Figure 1.**
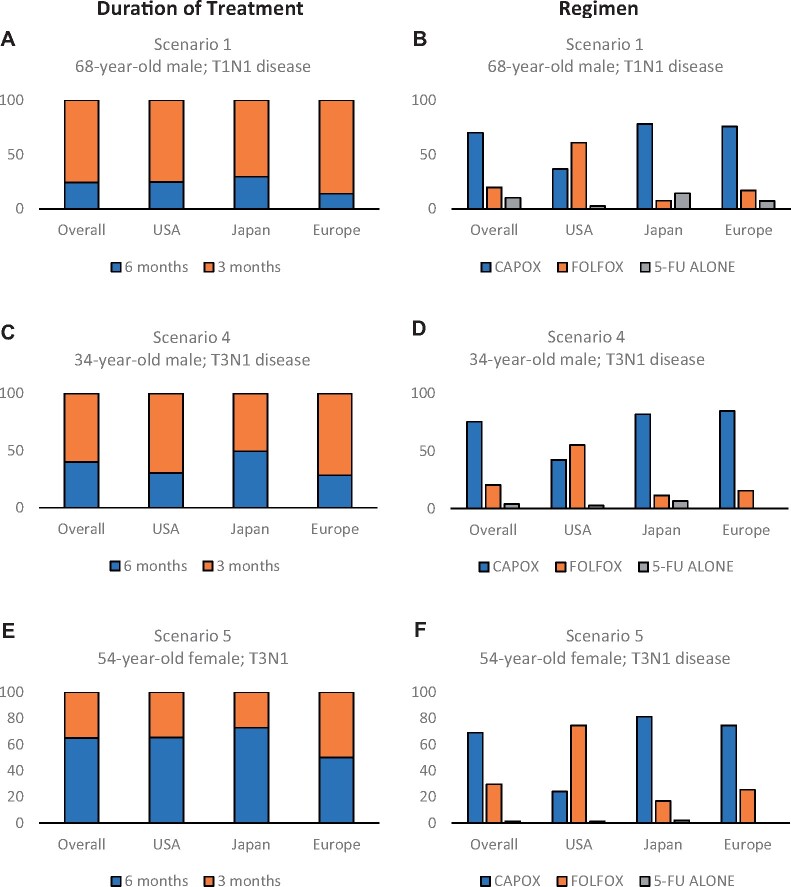
Results of the low-risk stage III scenarios by duration of treatment and regimen. The *y*-axis represents the percentages of patients. Descriptions of the scenarios are as follows: scenario 1: 68-year-old male with T1N1 colon cancer; scenario 4: 34-year-old male with T3N1 colon cancer; scenario 5: 54-year-old female with T3N1 colon cancer. Panels **A**, **C**, and **E** describe the duration of treatment chosen. Panels **B**, **D**, and **F** describe the treatment regimen chosen by survey respondents. CAPOX = capecitabine oxaliplatin; FOLFOX = 5-fluorouracil and oxaliplatin; FU = fluoropyrimidine; USA = United States.

***Low-Risk Stage III Patient Scenarios.*** Figure 1 indicates the regimen and duration of treatment chosen by clinicians for 3 scenarios describing patients with low-risk stage III colon cancer. Overall, for these 3 scenarios, there was a preference for 3 months (56.2%) rather than 6 months (43.8%) of treatment. This was most marked in Europe, where 68.8% of clinicians opted to use the shorter duration. In the United States, this figure was 59.7%, and in Japan, overall, fewer clinicians opted for 3 months (48.3%). The respondents from Japan showed the biggest variation in duration of treatment depending on the individual scenario. To treat a 68-year-old male patient with a moderately differentiated T1N1 tumor, 70.4% of Japanese respondents used 3 months of chemotherapy, whereas to treat a 34-year-old male patient with a poorly differentiated T3N1 tumor, only 27.2% opted to use the shorter duration.

Overall, most clinicians indicated they would prescribe CAPOX (70.3%) to treat low-risk patients, with fewer opting for FOLFOX (22.9%) or fluoropyrimidine (FU) alone (5.1%). There was an obvious variation in the regimen preference depending on the location of the respondents. Respondents from Japan (79.2%) and Europe (76.7%) consistently preferred CAPOX, whereas the majority of respondents from the United States chose FOLFOX for every scenario.

***High Risk Patient Scenarios.*** Four scenarios addressed clinicians’ approach to treating patients with high-risk stage III colon cancer. Combining the results of all 4 scenarios, overall, 87.6% of clinicians opted to give 6 months of treatment, and CAPOX was the preferred regimen (59.1%).

**Figure 2.**
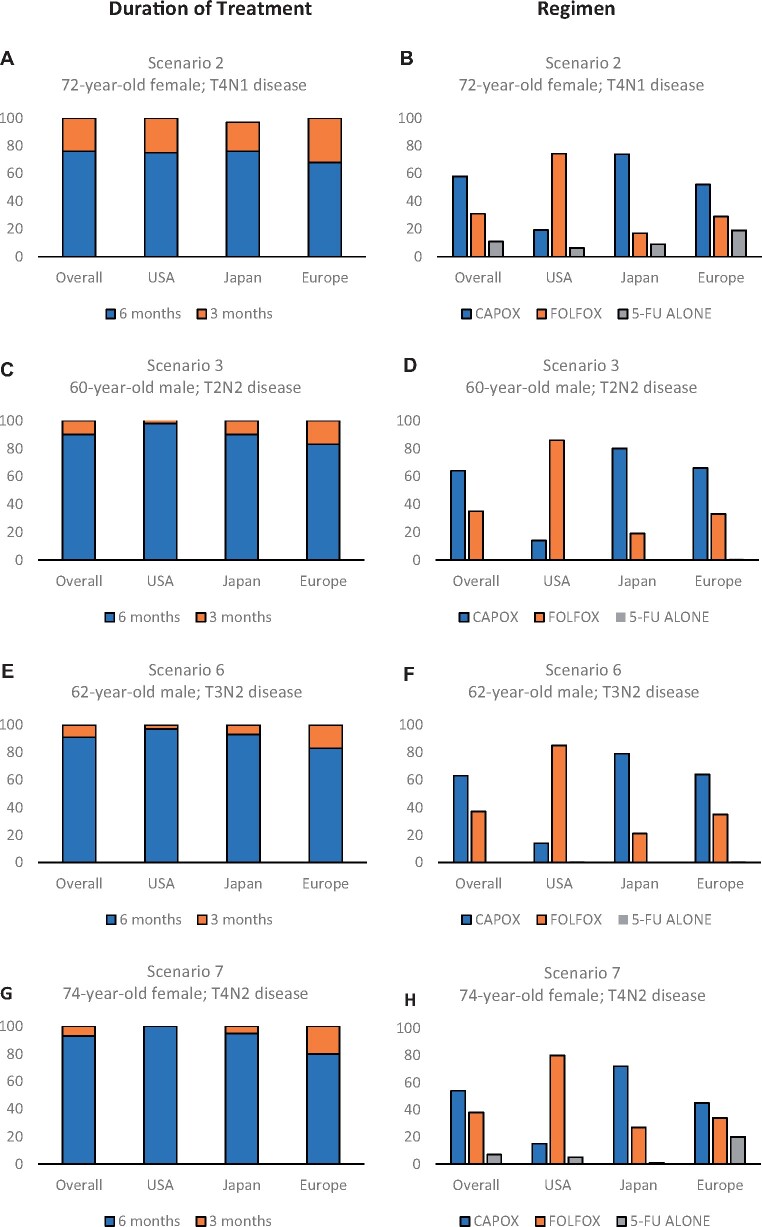
Results of the high-risk stage III scenarios by duration of treatment and regimen. The *y*-axis represents the percentages of patients. Descriptions of the scenarios are as follows: scenario 2: 72-year-old male with T4N1 colon cancer; scenario 3: 60-year-old male with T2N2 colon cancer; scenario 6: 62-year-old male with T3N2 colon cancer; scenario 7: 74-year-old female with T4N2 colon cancer. Panels **A**, **C**, and **E** describe the duration of treatment chosen. Panels **B**, **D**, and **F** describe the treatment regimen chosen by survey respondents. CAPOX = capecitabine oxaliplatin; FOLFOX = 5-fluorouracil and oxaliplatin; FU = fluoropyrimidine; USA = United States.

Figure 2 outlines the responses for each scenario by location of respondent. When approaching the treatment of a 72-year-old female patient with a moderately differentiated T4N1 tumor, the majority of respondents from all locations opted for 6 months of treatment (75.3% United States, 68.0% Europe, 79.1% Japan) (Figure 2, A). Participants from the United States preferred to use FOLFOX (74.4%), whereas Japanese respondents favored CAPOX (73.6%). The responses from the European group were divided: 52.0% CAPOX, 29.2% FOLFOX, and 18.7% FU alone (Figure 2, B).

The second high-risk scenario described a 60-year-old male with moderately differentiated T2N2 disease. For this patient, with N2 as the high-risk feature, a higher proportion of respondents chose to use 6 months of treatment (82.6% Europe, 90.2% Japan, 97.5% United States) (Figure 2, C), compared with the previous scenarios describing patients with T4N1 disease. Overall, 64.4% opted for CAPOX and 35.1% for FOLFOX. Again, the United States respondents favored FOLFOX (86.3%), those from Japan preferred CAPOX (80.4%), and the European group were more divided (66.3% CAPOX, 32.8% FOLFOX, 0.8% FU alone; Figure 2, D). The third high-risk scenario described a 62-year-old male with T3N2 moderately differentiated disease. The treatment decisions for this case were almost identical overall and by region compared with the responses given for a 60-year-old male with T2N2 disease.

**Figure 3.**
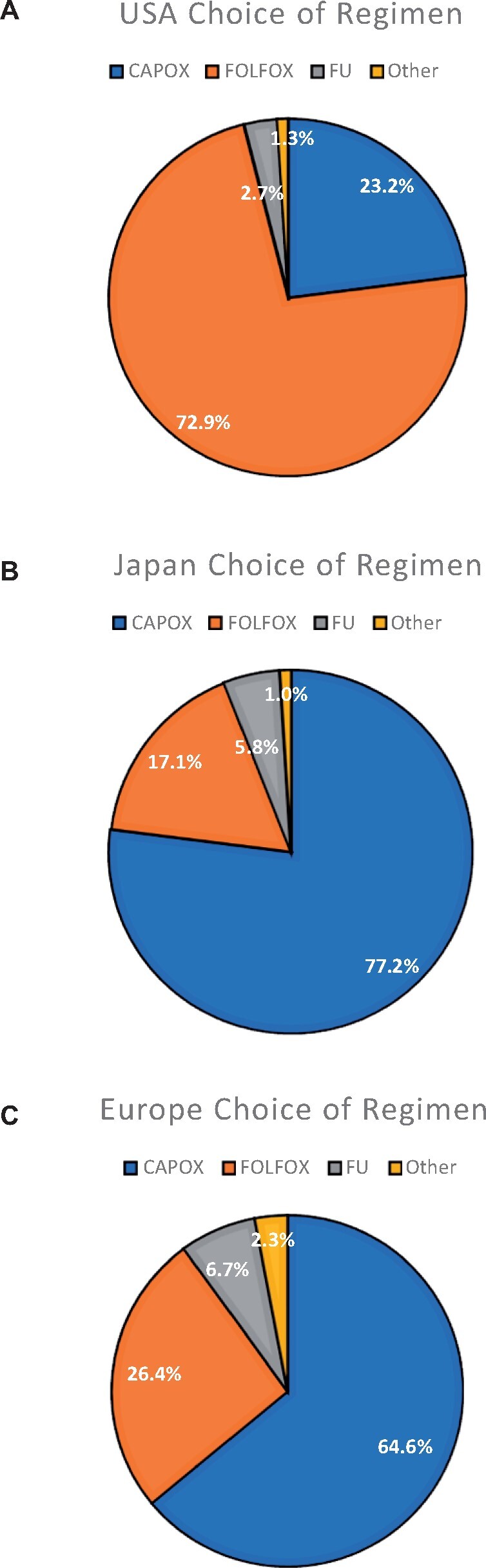
Choice of chemotherapy regimen according to location. The locations are as follows: **(A)** the United States, **(B)** Japan, and **(C)** Europe. CAPOX = capecitabine oxaliplatin; FOLFOX = 5-fluorouracil and oxaliplatin; FU = fluoropyrimidine; USA = United States.

High-risk scenario 4 was a 74-year-old female with high-risk features of T4 and N2 disease. Overall, 92.5% of clinicians opted to give 6 months adjuvant treatment (100.0% United States, 95.0% Japan, 80.4% Europe) (Figure 2, G), and there was a slight preference for CAPOX (54.5%) over FOLFOX (38.2%) and FU (7.4%) (Figure 2, H). When divided by location, the same pattern for previous high-risk scenarios was seen: clinicians in Japan chose CAPOX most frequently (72.1%), respondents from the United States preferred FOLFOX (79.7%), and in Europe, there were more varied responses (45.1% opted for CAPOX, 34.4% for FOLFOX, and 20.5% for FU alone). Figure 3 summarizes the choice of chemotherapy regimen chosen by respondents for all scenarios according to location.

## Discussion

The results of 6 international trials have offered new insights into possible differences between treatment regimen effects and risk stratification for patients with stage III colon cancer. It was not straightforward to predict how these results would shape clinicians’ views and prescribing practices or how quickly practice change may occur. This study addresses this gap by providing the largest survey of clinician opinion that has been conducted since the results from IDEA have been disseminated. This survey was conducted 4 months after the results were presented at ASCO and before the results appeared in peer-reviewed journals, and they were integrated into international guidelines for adjuvant treatment. Conducting a survey so soon may be a disadvantage if a study demonstrated that a new drug is beneficial as clinicians often have to wait until the drug is licensed (or reimbursed) before they can use it. In this case, as the decision for clinicians was about shortening treatment, an early survey has provided a unique insight that many clinicians would reduce treatment duration for some patients before publication of guidelines or of the trial in a peer-reviewed journal. The decision-making process here depends on several factors: the clinicians’ personal experience of the impact of persisting neuropathy for their patients, the study data showing poorer compliance with longer treatment, and the small difference in DFS with shorter treatment duration. The treatment duration would also be influenced by the chemotherapy regimen recommended and whether the patient had high- or low-risk stage III disease. These survey results show that prescribing changes can be made quickly before guidelines are published.

That the majority of clinicians (89.5%) considered giving 3 rather than 6 months of adjuvant treatment to some patients with stage III colon cancer was a departure from the previous standard treatment duration of 6 months and suggests an early shift in practice. Taking account of all responses for the 8 clinical scenarios, approximately one-third (31.0%) of responses indicated clinicians would prescribe 3 months of treatment. This implies that, although the IDEA collaboration did not meet its noninferiority target for 3 months, clinicians did feel that a shorter duration of treatment will be justified in certain circumstances, just 4 months after the IDEA results were disseminated. Individual scenario responses clearly showed that clinicians from all locations were more likely to use 3 months of treatment for low-risk disease. The novel risk stratification within stage III colon cancer has emerged from the IDEA collaboration, and the majority of respondents to this survey indicated that this division into low risk and high risk was useful and relevant.

Combining the responses for all of the stage III scenarios, respondents from Europe were most likely to change practice and prescribe 3 months of treatment (43.2%) compared with clinicians from Japan (24.7%) and the United States (29.8%). This may reflect a greater willingness to change practice based on the results of the SCOT trial, which mainly recruited patients from Europe and which did meet the overall population prespecified noninferiority test for 3 months vs 6 months of treatment (8). However, this was not the case for the IDEA-France, TOSCA, and HORG trials. Unfortunately, there were very few respondents from France, Italy, or Greece to explore differences between European countries in more detail.

The regimen preferences by country reflected the treatments used in the individual trials that contributed to the IDEA collaboration from these locations. For example, clinicians in the United States and Canada were more likely to prescribe FOLFOX (SWOG/CALGB 80702 used FOLFOX only), whereas those from Japan (in line with the ACHIEVE trial) and Europe (in line with the SCOT trial) were more likely to prescribe CAPOX. A greater contribution from clinicians from other countries in Europe may have altered this overall preference in the European cohort. For example, we know that in the TOSCA and IDEA-FRANCE trials, clinicians chose to use FOLFOX more commonly than CAPOX (64.0% and 90.0%, respectively) (11,12).

For scenarios describing patients with high-risk stage III colon cancer, N2 disease appeared more likely than T4 disease to influence respondents’ choice to give 6 months of treatment. This is an interesting finding considering that the forest plots from the IDEA collaboration did not show a difference between N1 and N2 disease when looking at duration of treatment, whereas for T4 disease, there was a non-statistically significant trend in favor of 6 months treatment. For patients with both T4 and N2 risk factors, very few respondents in this survey opted for 3 months of treatment (6.9%).

This survey has contributed to understanding the impact of the IDEA collaboration on clinician-prescribing practices soon after first publication of the IDEA results in abstract form. Other surveys of clinicians’ opinions have been performed (14,15) but with fewer respondents and from different locations. The strengths of this survey are the high number of responses and the timeliness with which it was disseminated after the IDEA collaboration results were publicized, only 4 months after the initial conference presentation. These results, therefore, provide formal documentation of the early reaction to the IDEA collaboration results.

One limitation of this survey is that distribution was performed using a list of investigators associated with the IDEA collaboration. Although these representatives were requested to distribute the survey widely, it is likely that many of the respondents recruited patientsto the IDEA collaboration trials. In addition, the majority of participants described themselves as working in an academic field, and a large proportion were from Japan (54.3%). Approximately one-third of respondents were surgeons, but these were predominantly from Japan. Because of a small number of medical oncologists in Japan, surgeons routinely prescribe chemotherapy, therefore their responses are representative of prescribing practices. There was a robust absolute response, the largest from any survey to date; however, because of the nature of the e-mail distribution lists used for dissemination, it was not possible to calculate a response rate, and there is no information on the potential respondents who received the survey but did not reply. As with any survey, there is the risk of response bias. Specifically, those who did reply may have been more aware of the IDEA collaboration results and more likely to change practice compared with nonresponders. Although it might be perceived as a limitation that the survey was performed prior to publication of the IDEA collaboration results in peer-reviewed journals and before many international guidelines were updated (16–18), as previously discussed, this may also be a strength of an early survey, as practice changes were easy to make without waiting for guidelines.

This survey provides insight into the early reaction of international practitioners to the results of an important collection of trials in the field of colorectal cancer treatment, which in combination recruited more than 12 000 patients worldwide. The survey has shown that within 4 months of the results of the IDEA collaboration being publicized, clinicians reported that they were prescribing 3 months of adjuvant treatment to patients with stage III colon cancer. In keeping with international guidelines published after this survey, clinicians were more likely to prescribe 3 months to patients if using CAPOX and for patients with low-risk disease (16–18). There was less consensus on the duration of treatment for patients with high-risk disease. The responses to the survey have indicated that the colorectal oncology community has broadly welcomed the new risk stratification for stage III colon cancer that emerged from the IDEA collaboration.

## Funding

This work was supported by grant (G0601705) from the SCOT study, by Medical Research Council (transferred to NETSCC—Efficacy and Mechanism Evaluation).

## Notes

**Role of the funder**: The funder funding the running of the SCOT study.

**Disclosures:** No authors have any conflicts of interest to declare.

**Author contributions:** Conceptualization—TI, JM. Survey design—TI, SZ, AL, JM. Analysis of data—all authors. Writing—Original draft—TI, CH, PI, JM. Writing—review and editing—all authors.

## Data Availability

The data underlying this article are available in the article.
